# Clinical outcomes and prediction of survival following percutaneous biliary drainage for malignant obstructive jaundice

**DOI:** 10.3892/ol.2014.1860

**Published:** 2014-02-07

**Authors:** GUANG YUAN ZHANG, WEN TAO LI, WEI JUN PENG, GUO DONG LI, XIN HONG HE, LI CHAO XU

**Affiliations:** Department of Radiology, Shanghai Cancer Center, Fudan University, Shanghai 200032, P.R. China

**Keywords:** malignant obstructive jaundice, percutaneous transhepatic biliary drainage, survival

## Abstract

The present study aimed to investigate the clinical outcomes of percutaneous transhepatic biliary drainage in patients with obstructive jaundice and identify potential predictors of patient survival. Clinical data from 102 patients (66 males and 36 females; median age, 63.50 years; range, 29–84 years) with a mean (± standard deviation) pre-drainage serum bilirubin level of 285.4 (±136.7 μmol/l), were retrospectively studied. Technical and clinical success, complications and survival time were recorded and their relationship with clinical factors, including age, obstruction level, liver metastases, serum bilirubin level and subsequent treatments following drainage, were analyzed by Fisher’s exact test. Patient survival rate and other predictors were analyzed by Kaplan-Meier survival curves and Cox’s proportional hazard model. The technical and clinical success rates were 100 and 76.5%, respectively. The presence of liver metastases was associated with reduced successful drainage. The overall complication rate was 7.8% and the overall median survival time was 185 days [95% confidence interval (CI), 159–211 days]. A log-rank test showed that age (χ^2^, 4.003; P=0.04), bilirubin levels following procedure (χ^2^, 5.139; P=0.02) and subsequent therapy (χ^2^, 15.459; P=0.00) affected survival time. However, Cox’s regression analysis revealed no administration of additional treatments to be a risk factor of survival (odds ratio, 2.323; 95% CI, 1.465–3.685; P=0.000). Percutaneous transhepatic biliary drainage for malignant biliary obstruction was found to be a safe and effective method to relieve jaundice caused by progressive neoplasms. Subsequent radical therapy following drainage, including surgery, chemotherapy and other local treatment types, are likely to increase patient survival.

## Introduction

Malignant obstructive jaundice is prevalent in periampullary carcinoma, cholangiocarcinoma, gallbladder carcinoma, and in metastatic lymph nodes of the hepatic hilum and hepatoduodenal ligament. In the majority of patients, malignant obstructive jaundice is incurable with a poor prognosis (1–3). As a result, biliary drainage is considered an important palliative treatment which relieves high serum bilirubin-related symptoms and provides patients with the opportunity to receive additional therapies, including surgery, chemotherapy and local treatment (4,5).

Percutaneous transhepatic biliary drainage (PTBD) and metallic stent insertion are established methods used for the relief of malignant biliary obstruction. Although the effectiveness of PTBD has been reported and discussed in a number of previous studies, predictors able to differentiate between a good and poor prognosis have not been established (6–8). Furthermore, the role of biliary drainage prior to subsequent treatment remains controversial. Notably, the possible benefits of preoperative biliary drainage (PBD) for cholangiocarcinoma and pancreatic cancer are debated (9,10). However, the potential beneficial effects of chemotherapy on patient survival following PTBD have been shown in several studies (11–13). Therefore, it is necessary to elucidate which features patients possess in order to ensure the maximum possible benefits for survival.

In the present study, 102 consecutive patients who had undergone PTBD and stenting were reviewed, with the aim to evaluate the technical and clinical success and complications of the procedure, and to analyze patient survival to identify potential prognostic factors.

## Materials and methods

### Patients

Between December 2009 and February 2011, 102 patients suffering from malignant obstructive jaundice received PTBD in the Department of Radiology (Shanghai Cancer Center, Shanghai, China). The clinical data of all patients were retrospectively studied. Patients or their families provided written informed consent.

### Data collection

Following approval by the internal review board, electronic clinical records of all patients were reviewed. Age, gender, primary cause of obstruction, obstruction level, Bismuth type, serum bilirubin levels prior to and following drainage, complications associated with intervention, additional treatments and survival, were analyzed. Technical and clinical success were recorded, in addition to any complications. Successful placement of the catheter into the correct position and bilirubin drainage, was taken to indicate technical success. Clinical success was defined by decreases in serum bilirubin levels of >20% within 7 days after drainage, compared with the bilrubin level prior to the procedure (14). Complications were divided into major and minor spectra according to the report standards and quality improvement guidelines for percutaneous transhepatic cholangiography, biliary drainage and percutaneous cholecystostomy from the Society of Interventional Radiology (15,16). Major complications included sepsis or cholangitis, hemorrhage requiring blood transfusion, abscess formation, peritonitis, cholecystitis, pancreatitis, pneumothorax, pneumonia, pleural infection and mortality. Minor complications included self-limiting hemorrhage, bilovenous fistulae and subcapsular biloma. Subsequent treatments included palliative surgery, systemic chemotherapy and transarterial chemoinfusion and embolization. Palliative surgery included Whipple-based regional pancreatectomy. The regimens applied in systemic chemotherapy were gemcitabine for pancreatic carcinoma and cholangiocarcinoma, and epirubicin plus cisplatin plus fluorouracil for gastric cancer. The chemoagents employed for transarterial chemoinfusion for hepatocellular carcinoma were doxorubicin and cisplatin.

Following intervention, the serum bilirubin levels of all patients were followed up on day 15 or later. For patients having received additional treatments, clinical records were tracked and telephone interviews were employed to check patient survival and general performance. Survival was calculated by the number of days since PTBD until mortality or until the study time limit of October 2012.

### Drainage procedure

PTBD and stenting were performed by at least two experienced interventional radiologists. Guided by sonography and X-ray fluorescence, an 8-French external pigtail nephrostomy catheter was placed proximal to the occlusion site to reduce damage to the liver parenchyma and tract system. Following a period of drainage, cholangiography was performed to investigate whether biliary wall edema was compromised by stent insertion. For patients with a life expectancy of <6 months, metallic stents were used following informed patient consent. MTN-DA self-expandable metallic stents [Micro-Tech (Nanjing) Co., Ltd, Nanjing, China] were used. Following successful stent placement, an 8-French external pigtail catheter was placed proximal to the stent for drainage and frequent flushing. The catheter was tapped after 2 days and was removed when proper drainage was confirmed, according to bilirubin levels and clinical findings.

### Statistical analysis

The Wilcoxon-signed rank test was used for comparison of changes in bilirubin levels prior to and following PTBD. A Student’s t-test was used to analyze the means between two groups. Fisher’s exact test was employed to compare clinical success rate stratified by clinical characters. Kaplan-Meier survival curves calculated the cumulative survival rates. Differences from curves were tested by the log-rank test. Univariate analysis was used to screen for potential candidate variables for multivariate analysis. Multivariate analysis was undertaken using the Cox’s proportional hazard model. All statistical analysis was performed using SPSS version 16.0 for Windows (SPSS Inc., Chicago, IL, USA). P<0.05 was considered to indicate a statistically significant difference.

## Results

### Patient characteristics

The study cohort consisted of 66 males and 36 females with a median age of 63.50 years (range, 29–84 years). The primary causes of jaundice included pancreatic carcinoma (n=48; 47.1%), carcinoma of the papilla of Vater and duodenal cancer (n=11; 10.8%), gallbladder tumor (n=8; 7.9%), cholangiocarcinoma (n=6; 5.8%), hepatocellular carcinoma (n=8; 7.8%), lymph node metastasis (n=15; 14.7%) and digestive tract invasion (n=6; 5.9%). In total, 18 (17.7%) patients developed proximal bile duct obstruction, which was categorized by Bismuth type as follows: Type I, n=15; type II, n=1; type III, n=1; type IV, n=1. In total, 37 (36.3%) patients received additional treatments following PTBD. Of these, 10 patients received surgery, 12 patients received chemotherapy and 10 patients received local therapy. In addition, 65 patients (63.7%) received symptomatic support treatments.

### Effectiveness of PTBD

Bile ducts were successfully drained in all patients with a 100% technical success rate. In the study group, 82 patients (80.4%) received PTBD only and metallic stents were inserted in 20 individuals (19.6%). Unfortunately, due to the limited number of stent cases, analysis of stent patency was abandoned.

Serum bilirubin levels were recorded prior to and following intervention. The mean baseline bilirubin level was 285.4±136.7 μmol/l (median, 267.3 μmol/l). After 7 days of drainage, levels fell to 192.0±128.5 μmol/l (median, 161.3 μmol/l). On day 15, mean bilirubin levels fell to 140.8±120.2 μmol/l (median, 106.5 μmol/l). The decrease in bilirubin levels pre- and post-procedure was statistically significant at the two time-points (Wilcoxon signed-rank test, P<0.001).

Overall clinical success was achieved in 78 cases, with a 76.5% success rate. In total, 20 patients (19.6%) exhibited a mild decrease and 4 patients (3.9%) experienced increases in the bilrubin level following PTBD. Among these, 3 patients succumbed to proximal bile duct obstruction. A total of 2 patients succumbed to cancer cachexia 53 and 28 days after PTBD. One patient succumbed to liver rupture caused by catheter displacement on day 18 after PTBD, having accidentally stretched the catheter out of place by ~5 cm which led to subsequent bleeding along the catheter.

However, in week 2 after intervention, a further 8 subjects achieved successful drainage. The relationship between successful drainage and clinical features was analyzed and is shown in Table I. Age, gender, obstruction level and serum bilirubin levels pre-intervention were not associated with successful drainage. However, the presence of liver metastasis was associated with a lower chance of clinical success (70.6 vs. 79.4%; P=0.033).

### Complications

A total of 2 patients succumbed to their conditions within 30 days of receiving PTBD. However, the patient who succumbed to invasive primary tumor progression was not included in data for major complications, as the cause of mortality was not drainage procedure-related. Minor complications occurred in 2 patients (1.9%), which presented as self-limiting hemorrhages and major complications were observed in 6 patients (5.9%). In total, 3 patients developed sepsis and presented with shivering and high fever during the 1–3- day period following catheter insertion. Hemoculture showed positive results in 2 cases and antibiotics against anaerobic bacteria were injected. The remaining patient with a negative blood culture experienced relief from the high fever and shivering 30 min after onset. One patient suffered from pancreatitis and received conservative therapy. The remaining 2 patients exhibited symptoms of pneumonia and pleural infection. Conservative therapy lasted for 3–5 days and was effective for the final 2 patients.

Thus, the overall complication rate was 7.8% (8/102 patients) and the procedure-related 30-day mortality rate was 0.9% (1/102 patients).

### Survival

By the cut-off date, 96 patients had succumbed to various conditions, 5 patients were still alive and 1 patient was lost to follow-up. The overall median survival time following PTBD was 185 days (95% CI, 159–211 days). The 6-month and 1-year survival rates were 43 and 14%, respectively.

Univariate analysis revealed three factors that significantly affected patient survival. These were patient age (χ^2^, 4.003; P=0.04), bilirubin levels following treatment (χ^2^, 5.139; P=0.02) and whether the patient had received additional treatment (χ^2^, 15.459; P=0.00). Cox’s regression results indicated that the absence of additional therapy was a risk factor for poorer survival following PTBD (Table II).

**Figure 1:**
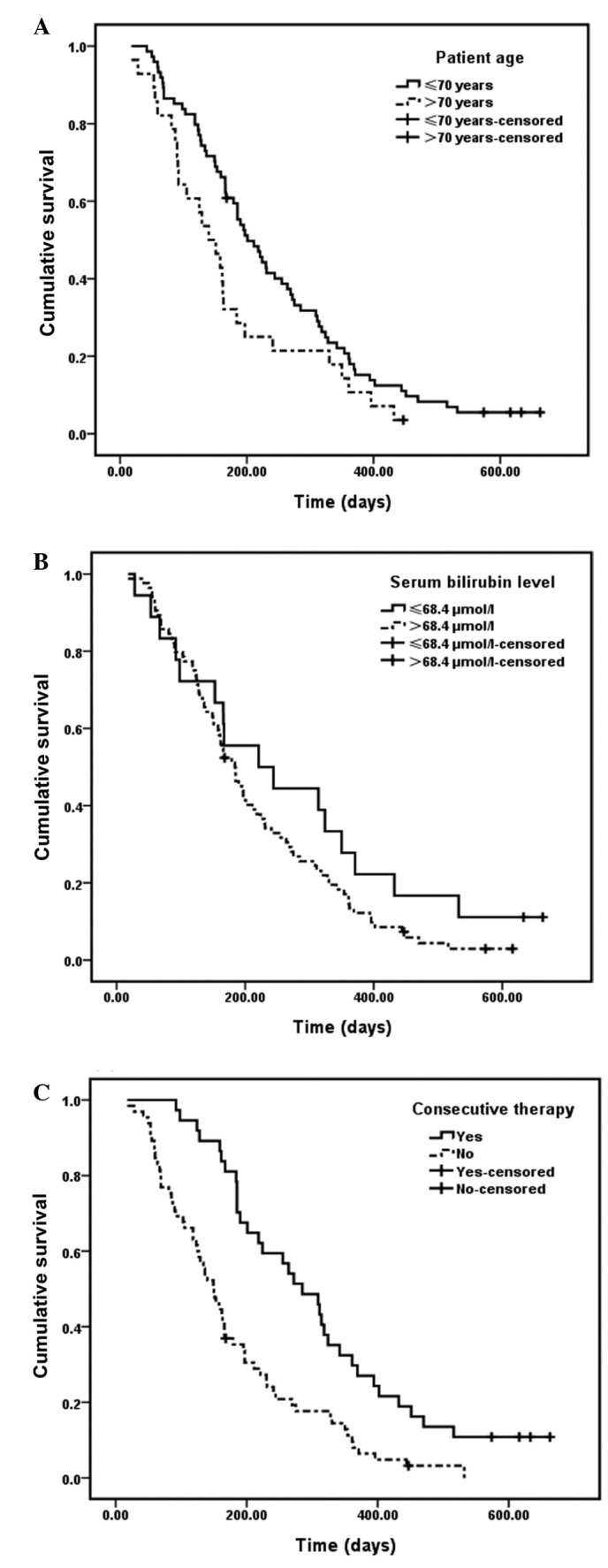
The differences of patient survival time according to several factors. Kaplan-Meier curves show: (A) A longer survival time following percutaneous transhepatic biliary drainage in patients of <70 years old; (B) A similar trend was observed in patients with serum bilirubin levels <68.4 μmol/l; (C) Patients receiving subsequent therapies, for example sugery, chemotherapy and other local types, demonstrated greater cumulative survival rates. Differences from curves were tested by the log-rank test and multivariate analysis was performed by Cox’s regression analysis.

The estimated median survival periods based on patient age of >70 or ≤70 years were 140 (95% CI, 97–183 days) and 201 days (95% CI, 161–241 days), respectively. Kaplan-Meier analysis showed that patients of >70 years old suffered from significantly shorter survival times following PTBD (P=0.04) (Fig. 1A).

Patients with post-drainage serum bilirubin levels ≤68.4 μmol/l had a median survival time of 244 days (95% CI, 166–322 days). In patients with serum bilirubin levels >68.5 μmol/l, the median survival time was 184 days (95% CI, 155–213 days). Log-rank survival analysis indicated a statistically significant difference between the two groups (serum bilirubin, ≤68.4 vs. >68.5 μmol/l; P=0.01) (Fig. 1B).

Patients receiving additional treatments, in the form of chemotherapy, palliative surgery and other local types, exhibited a significantly longer survival span of 285 days (95% CI, 218–352 days) compared with 150 days (95% CI, 123–177 days) for those without subsequent treatment (log-rank test, P=0.00) (Fig. 1C).

However, obstruction levels (P=0.46) and the presence of liver metastases (P=0.06) were not found to demonstrate a statistically significant relationship with patient survival.

## Discussion

Malignant biliary obstruction is often caused by external compression from lymph node metastases or internal stricture from neoplasms. Percutaneous biliary drainage and stenting are established and well reported methods used to relieve jaundice. Clinical success rates vary between 75 and 98% in various reports (17). In the present study, a slightly lower successful drainage rate (76.5%) was observed. The underlying cause may be the relatively higher base-line bilirubin levels of the present group. However, the bilirubin levels on day 15 after drainage showed a >20% decline in a further 8 patients, with an 84.3% clinical success rate. This is in accordance with previous literature.

Unsatisfactory clinical success rates have been found to be associated with liver metastases (18). Data of the current study consistently show that the presence of liver metastases is accompanied by a lower success rate for poor liver reserve and advanced systemic disease. However, patient age, high bilirubin levels prior to intervention and obstruction levels were generally not found to be associated with clinical success rate.

The complication rate of PTBD from previous studies ranges between 8 and 42% (19) and the 30-day mortality rate ranges between 2 and 19.8% (17,20). The overall complication rate (7.8%) and in-hospital mortality rate (0.9%) of the present study compared favorably to these. Antibiotics are not routinely applied prior to biliary procedures in our center due to a lack of reliable evidence in favor of their use (21). However, the incidence of sepsis (2.9%) in the present study is similar to data from Clark *et al,* in which all patients received prophylaxis (2%) (22).

The majority of malignant biliary obstruction patients suffered from a poor prognosis, due to advanced metastases and/or a poor general health status. A 185-day median survival time was observed in the present study, which appeared longer than intervals of 79–104 days reported in previous studies (13,23,24). By contrast, participants of the present study received chemotherapy, surgery, transarterial chemoinfusion and embolization, which may account for the prolonged survival rates observed. There have been specific potential predictors discussed in previous literature, including patient age, performance status, tumor histology type, obstruction level, liver metastasis, serum bilirubin level following PTBD and chemotherapy following drainage. However, results are controversial. Unlike the results of Migita *et al* (13) and Gwon *et al* (24), with bilrubin levels of 2 mg/dl, the present study the present study observed bilrubin levels of 68.4 μmol/l (4 mg/dl). This contradiction may have arisen due to three factors. Firstly, study subjects presented a heterogeneous group of diseases, among which the progressiveness is complex. Secondly, as described, the baseline levels of the present study group are relatively high (285.4 vs. 145 and 172.7 μmol/l; the present study vs. the results of Migita *et al* (13) and Gwon *et al* (24), respectively). Therefore, 7 days may not be long enough for patient bilirubin levels to return to a lower level. Log-rank analysis of serum bilirubin levels 2 weeks after drainage revealed a significantly longer survival time [244 (median overall survival time in patients with bilrubin levels <4 mg/dl) and 166 days (median overall survival time of patients with bilrubin levels >4 mg/dl); 95% CI, 190–298 and 140–192 days, respectively; P=0.007) in patients with bilirubin levels returning to <4 mg/dl. Finally, additional treatments administered to either group of patients were comparable (35 vs. 44% for bilirubin levels >68.4 μmol/l and ≤68.4 μmol/l, respectively; Fisher’s exact test, P=0.432). Thus, additional therapies may prolong patient survival time, regardless of the degree by which the post-drainage bilirubin level is reduced.

High serum bilirubin levels often provide contraindications for surgery, chemotherapy, radiotherapy and local methods, including transarterial chemoembolization and radio frequency ablation for poor liver reserve. A reduction in bilirubin levels following PTBD offers the possibility for patients to receive radical antitumor therapies. However, patients with high bilirubin levels should only receive supportive care. (4,25). The importance of additional therapies on survival is highlighted in the present study, as previously documented. Migita *et al* (13) observed a prolonged survival period in patients with metastatic gastric cancer who received chemotherapy following PTBD, and chemotherapy was observed to be tolerable and associated with an acceptable quality of life. However, the necessity of PBD has been queried by numerous studies, including a multicenter, randomized trial (26). This concluded that PBD increases post-surgery complications in pancreatic head cancer patients. However, the debate remains. Considering that an endoscopic method was used for the trial, the percutanous pathway may alternatively be analyzed. Furthermore, considering that surgery complications were evaluated, other aspects may be analyzed, for example mortality and survival time. Percutaneous drainage has been recommended in a recent study for PBD (27). PBD showed no effect on the mortality rate in jaundiced patients with hilar cholangiocarcinoma (28). In the present study, a markedly increased survival time was observed in patients having received surgery following biliary drainage. However, patients having received subsequent treatment exhibited a good performance status and relatively fewer advanced tumors. These imbalanced clinical backgrounds may affect analysis of survival times. Therefore, randomized control trials are essential for evaluating the potential benefits of successive treatment on survival.

The present study undoubtedly holds certain limitations, including the retrospective design and the heterogeneity of primary tumors. In addition, the effect of various treatment methods on survival rate were mixed. Thus, types which are ‘harmful’ to survival may not be exposed. However, this issue may be addressed in future studies. In conclusion, PTBD is a safe and effective way to relieve jaundice caused by malignant tumors. The utilization of subsequent radical therapies following drainage is likely to increase patient survival.

## Figures and Tables

**Table I:** Univariate analysis of the relationship between successful clinical drainage and patient characteristics.

	Bilirubin, n (%)		
		
Features	≥20% decrease	Increase or minor change	P-value
Age, years			
>70	19 (67.9)	9 (32.1)	0.295
≤70	59 (79.7)	15 (20.3)	
Gender			
Male	46 (69.7)	20 (30.3)	0.052
Female	32 (88.9)	4 (11.1)	
Obstruction level			
Hilar	13 (72.2)	5 (27.8)	0.760
Non-hilar	65 (77.4)	19 (22.6)	
Liver metastases			
Present	24 (70.6)	10 (29.4)	0.033
Absent	54 (79.4)	14 (20.6)	
Bilirubin prior to PTBD, μmol/l			
>342	26 (81.2)	6 (18.8)	0.616
≤342	52 (76.5)	18 (23.5)	

PTBD, percutaneous transhepatic biliary drainage.

**Table II:** Multiple Cox regression analysis of factors independently associated with survival following PTBD.

Variables	OR	95% CI	P-value
Age ≤70 years	1.263	0.734–2.171	0.399
Serum bilirubin ≤68.4 μmol/l following PTBD	1.215	0.797–1.853	0.365
Recieved successive therapies	2.323	1.465–3.685	0.000

PTBD, percutaneous transhepatic biliary drainage; CI, confidence interval.
